# Clonal Hematopoiesis and Inflammatory Pathways in Heart Failure with Preserved Ejection Fraction

**DOI:** 10.1007/s11897-026-00779-4

**Published:** 2026-10-13

**Authors:** Eline Verghote, Vanessa P. M. van Empel, Job A. J. Verdonschot

**Affiliations:** 1 Department of Cardiology, Cardiovascular Research Institute Maastricht, Maastricht University, Maastricht, the Netherlands; 2 Department of Clinical Genetics, Maastricht University Medical Center+, Maastricht, the Netherlands; 3 Debyelaan 25, Maastricht, 6229 HX Netherlands

**Keywords:** HFpEF, inflammation, CHIP, incidence, prognosis

## Abstract

**Purpose of Review:**

This review aims to examine the role of clonal hematopoiesis of indeterminate potential (CHIP) in the pathophysiology of heart failure with preserved ejection fraction (HFpEF), focusing on its contribution to inflammation, disease development, and clinical implications.

**Recent Findings:**

Recent studies demonstrate that CHIP is enriched in HFpEF populations and is associated with increased disease risk and worse clinical outcomes. Mechanistically, CHIP promotes inflammatory signaling through activation of pathways such as the NLRP3 inflammasome and IL-1β/IL-6 axis. TET2 mutations, in particular, have been consistently linked to diastolic dysfunction and adverse remodeling, especially in patients with high inflammatory burden and cardiometabolic comorbidities.

**Summary:**

CHIP represents a novel link between ageing, inflammation, and HFpEF, contributing to disease heterogeneity and progression. It may serve as a biomarker for early detection, risk stratification, and patient phenotyping, while also offering potential therapeutic targets. Future research should focus on validating these findings and integrating CHIP into precision medicine approaches for HFpEF.

**Graphical Abstract:**

**Figure:**
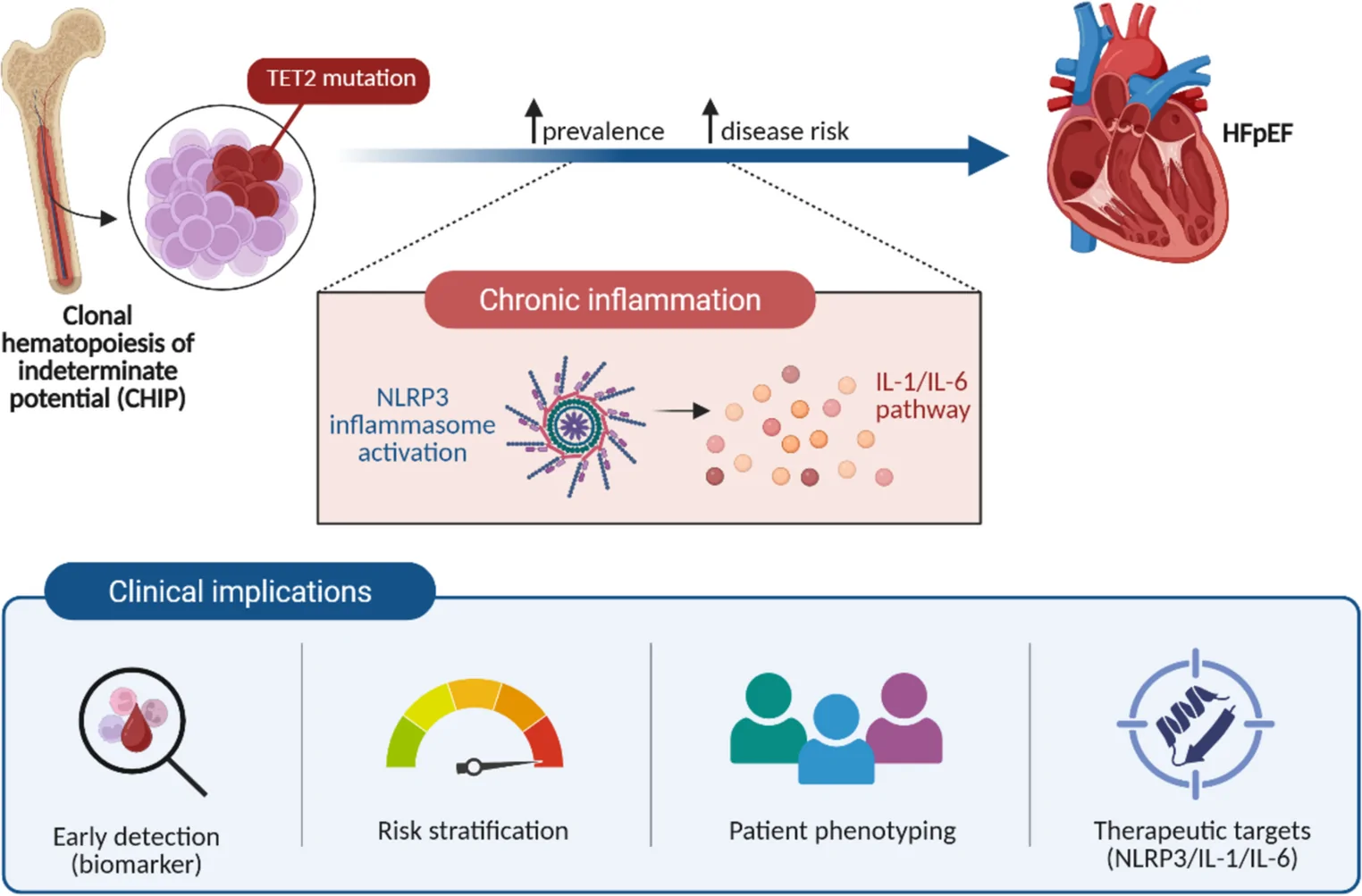
Clonal hematopoiesis as a driver of chronic inflammation in heart failure with preserved ejection fraction. HFpEF: heart failure with preserved ejection fraction

## Introduction

Heart failure with preserved ejection fraction (HFpEF) is a prevalent and challenging form of heart failure, particularly in ageing populations [1]. Despite its growing clinical impact, effective disease-modifying therapies remain limited, reflecting the incomplete understanding of its underlying pathophysiology. HFpEF is increasingly recognized as a heterogeneous syndrome driven by a complex interplay of comorbidities, including obesity, hypertension, diabetes, and ageing [2, 3]. However, systemic inflammation is a unifying concept in all these diseases driving HFpEF development and progression [4]. Chronic low-grade inflammation is thought to promote endothelial dysfunction, myocardial fibrosis, and impaired ventricular relaxation, all of which contribute to the HFpEF phenotype. This paradigm has shifted the focus from purely hemodynamic abnormalities to broader multisystem processes, highlighting inflammation as a potential therapeutic target [5, 6]. In this context, clonal hematopoiesis of indeterminate potential (CHIP) has recently emerged as a novel link between ageing, inflammation, and cardiovascular disease [7]. CHIP is characterized by the age-related acquisition of somatic mutations in hematopoietic stem cells, leading to clonal expansion of mutated blood cell populations. These clones have been associated with increased inflammatory signaling and a heightened risk of cardiovascular events, independent of traditional risk factors [8]. For an in-depth description of the underlying mechanistic pathways we refer to previous reviews on this topic. Growing evidence suggests that CHIP may contribute to the inflammation underlying HFpEF. Experimental and clinical studies indicate that CHIP-associated mutations can drive maladaptive immune responses, potentially accelerating myocardial remodeling and functional impairment [9]. However, the precise mechanisms linking CHIP to HFpEF remain incompletely defined. The clinical implications of this association are still being elucidated. Importantly, CHIP is not a single biological entity: its inflammatory and cardiovascular consequences appear to depend on the mutated gene, mutation type, clone size and disease context. The focus of the review is on the link between CHIP, inflammation and HFpEF from a clinical perspective, summarizing current evidence and discussing its potential utility as a biomarker and therapeutic target in HFpEF.

## Inflammation in HFpEF

HFpEF arises from the cumulative impact of ageing and a high burden of cardiometabolic and systemic comorbidities such as hypertension, obesity, type 2 diabetes (T2D), chronic kidney disease, and atrial fibrillation [2, 10]. A key concept in HFpEF is that inflammation reflects chronic metabolic and hemodynamic stress induced by these coexisting conditions. Although each comorbidity activates the immune responses through distinct mechanisms, they share the ability to promote persistent immune activation. Consequently, elevated circulating inflammatory mediators are commonly observed in HFpEF patients that contributes to myocardial dysfunction and adverse cardiac remodeling [5, 11].

A mechanistic pathway linking inflammation to myocardial dysfunction involves impaired nitric oxide (NO) signaling. Pro-inflammatory cytokines and oxidative stress reduce the bioavailability of endothelial NO, thereby disrupting microvascular homeostasis. Endothelial activation further promotes leukocyte recruitment into the myocardium, driving fibroblast activation and extracellular matrix deposition [5, 12]. In parallel, impaired endothelial NO signaling dysregulates the NOS/NO-sGC-cGMP-protein kinase G axis in cardiomyocyte [6]. Protein kinase G -mediated phosphorylation of titin is crucial for myocardial compliance, and reduced protein kinase G activity results in increased cardiomyocyte stiffness and diastolic dysfunction [13, 14].

Multiple inflammatory mediators have been identified in HFpEF. Circulating IL-6 levels are frequently elevated in HFpEF, with particularly high concentrations observed in patients with obesity and T2D [15, 16]. Furthermore, increased IL-6 levels have been linked to incidence disease as well as to poor prognosis, including increased risk of death or heart failure-related hospitalization [17, 18]. One of the downstream effects of IL-6 involves the upregulation of hepatic C-reactive protein (CRP), a widely used clinical biomarker of systemic inflammation [19]. Elevated CRP levels have similarly been associated with increased cardiovascular risk and all-cause mortality in HFpEF. Moreover, CRP levels tend to rise progressively in HFpEF with increasing comorbidity burden and are particularly linked to obesity and atrial fibrillation [11, 20]. Beside IL-6 signaling, IL-1β also contributes to the inflammatory landscape of HFpEF. This marker is a key mediator of the innate immune activation regulated by the NLRP3 inflammasome, and has been associated with rehospitalization risk and HFpEF progression [21]. Importantly, IL-1β appear to play a role in pulmonary hypertension, a frequent condition of HFpEF. Experimental models of pulmonary hypertensive HFpEF have demonstrated upregulation of IL-1β in pulmonary macrophages and inhibition attenuated pulmonary hypertension development [22, 23]. The pro-inflammatory cytokine TNF-α has also been implicated in HFpEF. Circulating TNF-α levels are elevated compared to healthy control [24] and have been associated with incident HFpEF and adverse clinical outcomes [18, 25]. In addition, TNF-α signaling has been linked with low glomerular filtration rate, hypertension, T2D and atrial fibrillation [24], further supporting the concept that inflammation in HFpEF is largely driven by its comorbidities.

HFpEF patients do not display a uniform inflammatory signature. Distinct immune profiles can be observed depending on the preexisting comorbidities. Obesity-related HFpEF is characterized by “meta-inflammation” [26], driven by adipose tissue expansion and immune cell accumulation, whereas hypertension and T2D are more associated with endothelial inflammation and oxidative stress [27, 28]. Age and gender exert additional modulatory effects on immune responses. Aging is accompanied by an increase in inflammatory responses, often referred to as “inflammaging”, and may in part be driven by ageing-associated clonal hematopoiesis of indeterminate potential (CHIP) [29, 30]. Furthermore, hormonal changes predispose women to metabolic dysregulation and heightened inflammatory risk.

Collectively, inflammation in HFpEF is a multifactorial process arising from comorbidity-driven systemic inflammation, endothelial dysfunction, and myocardial immune cell infiltration. The heterogeneity of inflammatory profiles in HFpEF underscores that immune activation reflects the interplay between age, sex, comorbidity burden and underlying molecular drivers, contributing to differences in disease severity, progression, and therapeutic response (Table 1). A deeper understanding of the immunomodulatory mechanisms that drive HFpEF is therefore essential for better risk stratification and the development of personalized medicine.

**Table 1:** Overview of inflammatory pathways and their clinical evaluation

Pathway	Agent	Clinical trial	Outcome
NO signaling	Sildenafil; Inorganic nitrite	RELAX; INDIE-HFpEF	No improvement in exercise capacity or clinical status in HFpEF patients
IL-1β signaling	Anakinra	D-HART; D-HART2	Improvement in peak oxygen consumption in HFpEF patients; No improvement in cardiorespiratory fitness in HFpEF patients
NLRP3 inflammasome	Colchicine	LoDoCo2; COLHEART-PRESERVED	Reduced risk of cardiovascular events in patients with chronic coronary disease; Ongoing in HFpEF
IL-6 signaling	Ziltivekimab	HERMES	Ongoing in HFpEF
TNF-α signaling	Infliximab; etanercept	ATTACH; RENEWAL	No improvement in clinical endpoints in HFrEF patients Translational potential in HFpEF remains unproven

## Inflammation and chip

Clonal hematopoiesis (CH) is defined by the presence of an expanded population of clonal hematopoietic stem and progenitor cells in individuals without a diagnosed hematologic malignancy [31]. Clonal hematopoiesis of indeterminate potential (CHIP) refers to a subset of CH characterized by leukemia-associated somatic mutations with a variant allele frequency of at least 2%, in the absence of cytopenia, dysplasia, or overt neoplasia. This variant allele frequency corresponds to more than 4% circulating blood cells originating from a single clone, assuming heterozygosity of the mutation. CHIP is increasingly recognized as a mechanistic driver of chronic, low-grade inflammation through its impact on innate immune-cell function and cytokine signaling [7]. The best characterized inflammatory mechanism is the upregulation of the inflammasome, although this is mutation-dependent. For example, in murine models of atherosclerosis, TET2-deficient macrophages show increased IL-1β transcription and NLRP3 inflammasome activation, whereas JAK2^V617F^ macrophages have been associated with enhanced AIM2 inflammasome activation, resulting in distinct downstream phenotypes [32–34]. The detrimental effects in these murine models of TET2-related CHIP could be reversed using NLRP3-inflammasome inhibition [34]. There are multiple sources of evidence that link CHIP to inflammasome activity in the heart, mainly guided by evidence coming from *TET2* disease models [9]: (1) inhibition of the NLRP3 inflammasome improves cardiac function in a *Tet2* CHIP murine model with HFrEF [35]; (2) genetic deficiency of the NLRP3 inflammasome attenuated diastolic dysfunction in a *Tet2* CHIP murine model with HFpEF [36]; (3) canakinumab leads to a larger reduction in major adverse cardiovascular events in patients with hematopoietic *TET2* mutations [37]; and (4) colchicine reduces the risk for myocardial infarction in carriers of *TET2* mutations [38]. Although this establishes the role between CHIP and cardiac activity, the latter two studies do not relate to heart failure. A causal link between CHIP and inflammation has been demonstrated across multiple disease models and patient populations, indicating that this association is not disease-specific. This broad relevance makes CHIP-driven inflammation an appealing therapeutic target in a range of cardiovascular diseases, although its clinical benefit remains to be established for each condition individually.

## Current Evidence of Clonal Hematopoiesis in HFpEF

The link between CHIP and cardiovascular disease was first established a decade ago, when large-scale sequencing studies associated age-related somatic mutation with increased mortality and coronary heart disease risk [8, 39]. Complementary experiments using TET2-deficient bone marrow transplantation in mice demonstrated accelerated atherosclerosis development driven by enhanced inflammasome-mediated inflammation [8, 34]. Since then, CHIP has gained attention as a modifier of cardiovascular risk and may contribute to disease susceptibility and progression in heart failure [7, 40]. Although most studies focused on the association between CHIP and (ischemic) HFrEF, recent clinical studies have evaluated the prevalence and prognostic relevance of CHIP in HFpEF populations and consistently report enrichment of CHIP compared with control individuals without heart failure. Moreover, CHIP mutations were significantly associated with a 42% increased risk to develop HFpEF [41].

Mouse models of HFpEF remain challenging, as the condition reflects a complex and multifactorial pathophysiology in humans [42]. Nevertheless, such models are valuable for establishing causal relationships between CHIP and disease development. To date, two studies have explored the role of CHIP in HFpEF using murine models (Table 2) [36, 43]. In these studies, HFpEF was induced by a combination of a high-fat diet (HFD) and inhibition of nitric oxide synthesis with L-NAME. Mice receiving *Tet2*-deficient bone marrow transplantation developed more pronounced cardiac hypertrophy, diastolic dysfunction, and fibrosis compared to controls [43]. Notably, HFD/L-NAME exposure led to significant clonal expansion specifically in the *Tet2*-deficient mice, while *Tet2* deficiency alone was sufficient to increase circulating inflammatory cells. In a complementary model, hematopoietic-specific *Tet2* knockout mice subjected to HFD/L-NAME exhibited worsened diastolic function [36]. Transplantation of *Tet2*-deficient bone marrow into wild-type recipients reproduced this phenotype following HFpEF induction, confirming a hematopoietic-driven effect. In contrast, similar experiments using *Dnmt3a*-deficient bone marrow did not result in impaired diastolic function, indicating genotype-specific differences. Mechanistically, intracardiac myeloid cells from *Tet2*-deficient mice showed upregulation of inflammatory pathways, including NF-κB signaling pathway and TGF-β signaling. This inflammatory signature was largely driven by expansion of a pathogenic and profibrotic Spp1⁺ macrophage subset. Both studies suggest a causal role between *TET2* mutations and the development of HFpEF-associated disease.

**Table 2:** Overview of mouse models investigating the causal effects of clonal hematopoiesis in HFpEF models

	CHIP induction	HFpEF trigger	Cardiac effects
Cochran et al.	Tet2-deficient BMT	HFD + L-NAME	Increased cardiac hypertrophy Worse diastolic function Increased cardiac fibrosis Expansion of *Tet2*-deficient cells
Pandey et al.	Hematopoietic cell-specific Tet2 KO	HFD + L-NAME	Worse diastolic function
	Tet2-deficient BMT		
	Dnmt3a-deficient BMT		No effect

**Fig. 1:**
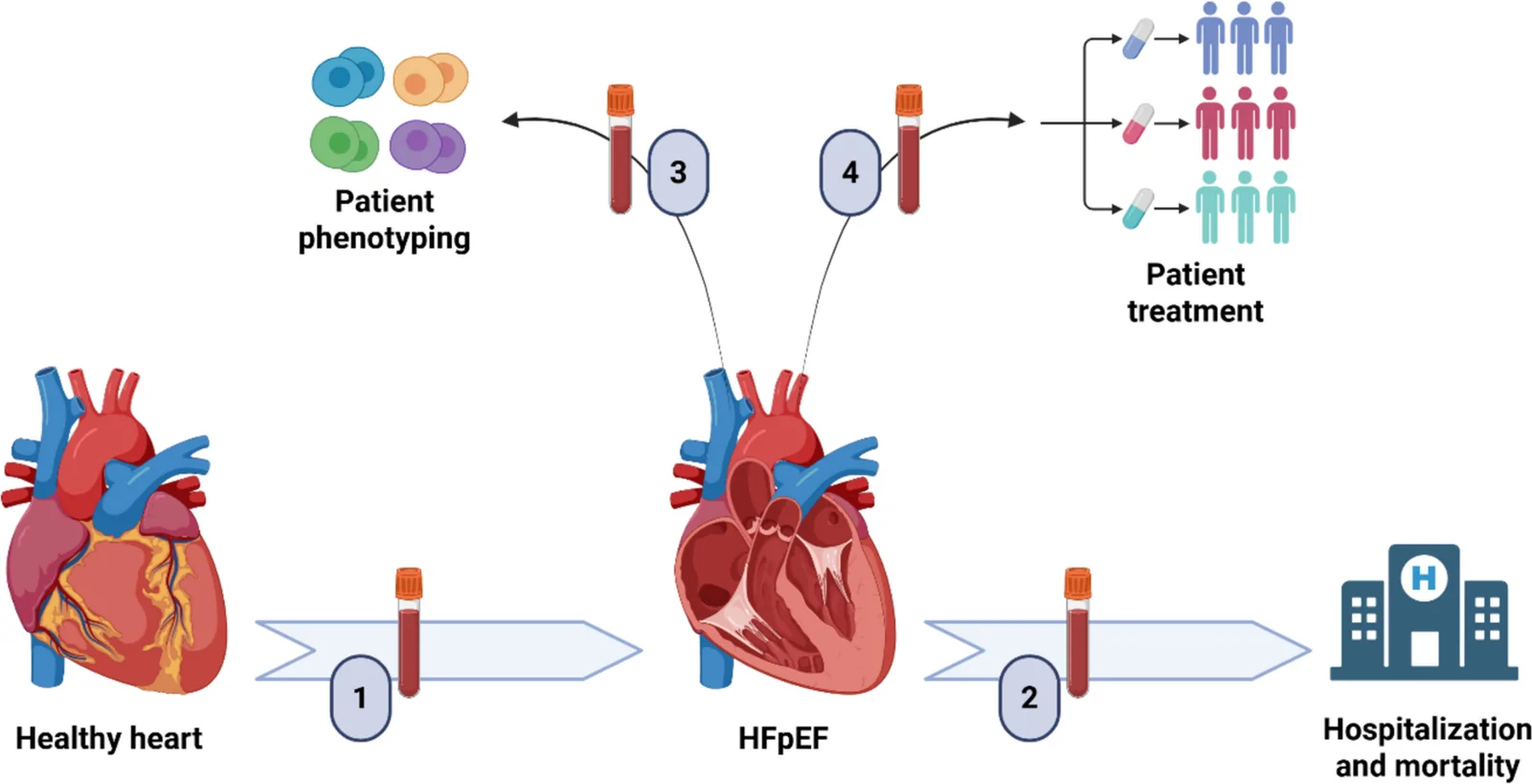
Potential role of clonal hematopoiesis to act as a biomarker. CHIP may function as a biomarker for: (1) the early detection in the development of heart failure with preserved ejection fraction (HFpEF), (2) risk stratification for patients with HFpEF, (3) the identification of patient subgroups within the heterogeneous HFpEF population, and (4) determining a treatment plan for a patient and monitor treatment response

**Fig. 2:**
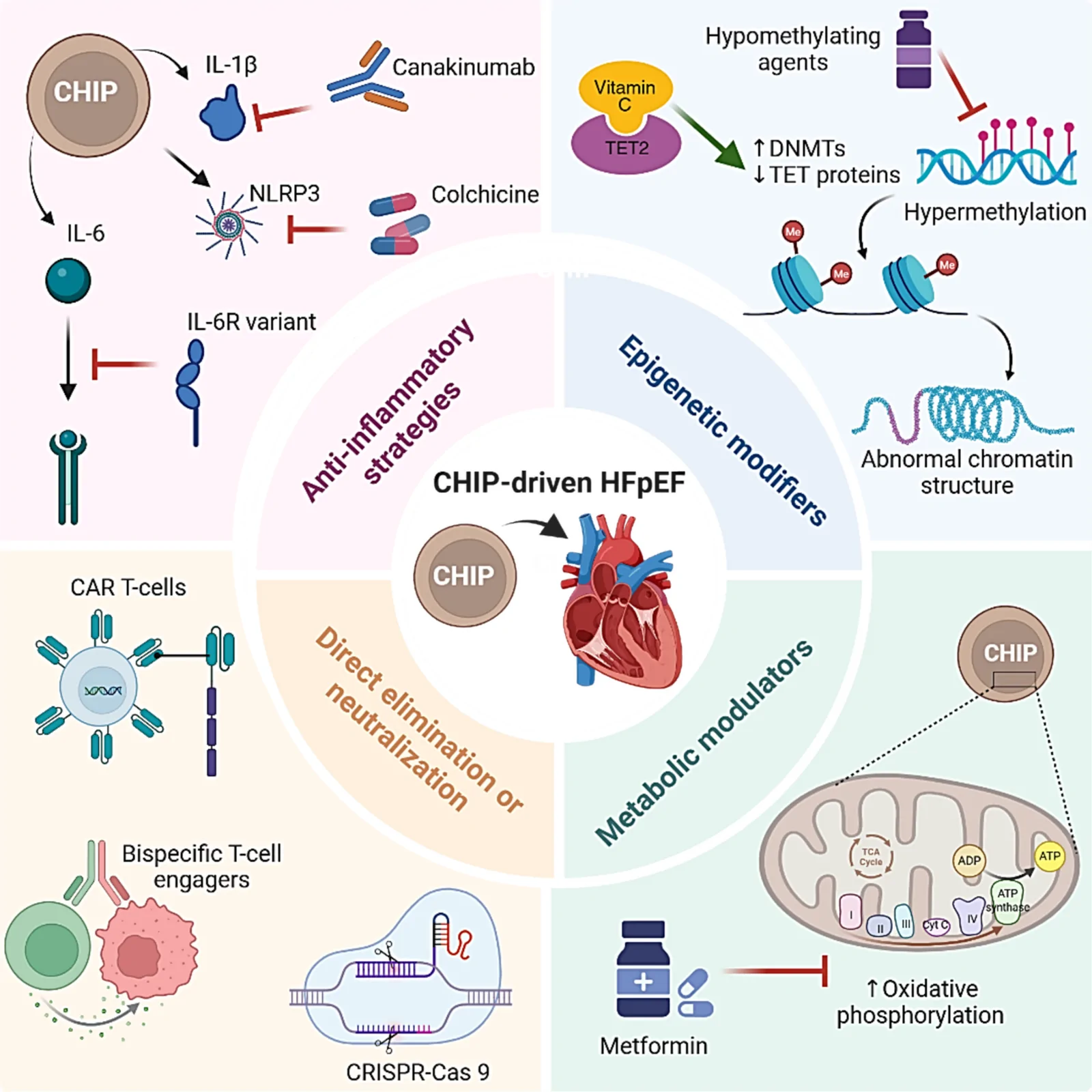
Therapeutic approaches to target clonal hematopoiesis in HFpEF. CHIP may serve as a modifiable therapeutic target in HFpEF. Anti-inflammatory strategies (canakinumab, colchicine, IL6R variant) include targeting IL-1β, IL-6 and NLRP3 signaling. Epigenetic modifiers (hypomethylating agents, vitamin C) restore normal differentiation of mutant hematopoietic cells through modulation of DNA methylation and TET2 activity. Metabolic interventions, such as metformin, increase metabolic respiration. Direct elimination or neutralization of mutant clones involves immune-based approaches (CAR T-cells, bispecific T-cell engagers) and genome editing techniques (CRISPR-Cas 9)

Human studies are divided into population cohorts investigating the association of CHIP and the incidence of HFpEF, and patient cohorts that investigate the effect of CHIP on prognosis. Collectively, studies show an association between CHIP and HFpEF development with a hazard ratio (HR) of ~ 1.4 (Table 3). Gene-specific analyses indicate that TET2 mutations are enriched in HFpEF, with an approximately 2.4-fold higher prevalence compared to those without HFpEF [31, 43]. These mutations are further associated with an increased risk of HFpEF development [30, 44]. One studied investigated specifically the potential link with inflammation in disease development, and stratified patients by levels of C-reactive protein (CRP) [45]. The association between CHIP and HFpEF was observed only in individuals with elevated CRP levels (≥ 2 mg/L), while no increase in cumulative incidence was seen in those with CHIP and lower CRP (< 2 mg/L). These findings suggest that CHIP may increase HFpEF risk primarily in the presence of systemic inflammation. Although composite DNMT3A CHIP has shown less consistent associations across studies, mutation-specific effects, particularly for the DNMT3A R882 variant, have been linked to incident HFpEF [30], suggesting that the cardiovascular implications of CHIP may be gene, but also mutation specific. The association between CHIP and HFpEF is modified by both age and systemic inflammation. Indeed, Ezzat et al. demonstrated that CHIP mutations are linked with a higher incidence of HFpEF in older females [30]. In addition, HFpEF patients with an age ≥ 70 and in the presence of *TET2* or *DNMT3A* mutations showed a markedly increased risk of cardiovascular-related hospitalization [43]. Also in a more recent study, CHIP was associated with a two-fold increase in risk of heart failure hospitalization or death [36]. A higher number of CH mutations and a higher burden of CH were also associated with worse clinical outcomes. However, CHIP is not exclusively relevant in elderly populations. In individuals younger than 65 years, CHIP has also been associated with HFpEF incidence, which was mainly driven by DNMT3A mutations. Therefore, early identification of CHIP could improve risk stratification and preventive strategies in HFpEF [7, 45]. Although these findings need to be validated in external cohorts and different populations, it seems that mainly *TET2* is associated with the incidence of HFpEF and influence the prognosis of the diseases, independent of clone size (Figs. 1 and 2).

**Table 3:** Overview of clinical studies investigating the association of clonal hematopoiesis with the development or prognosis of HFpEF

Study	Sample size	Sequencing strategy		Association (hazard ratio)	Specific gene associated with HFpEF
Shi et al.	374	Targeted	Incidence	1.1 [0.7–1.8]*	*DNMT3A*,* TET2*
Ezzat et al.	6677	Targeted		1.2 [0.9–1.5]	*TET2*‡
Schuermans et al.	8090	WGS		1.3 [0.9–1.8]	*TET2†*
Reiner et al.	5214	WGS		1.4 [1.1–1.9]	*TET2#*
Cochran et al.	117	Targeted	Prognosis	5.1 [1.1–24.2]	*TET2*
Pandey et al.	429	Targeted		2.07 [1.01–4.22]	*TET2*

* Association was significant in the sub-cohort of patients younger than 65 years: HR 2.1 [1.01–4.4]† Association was significant for TET2: HR 2.4 [1.3–4.1]‡ Association was significant for TET2: HR 1.4 [1.03–1.9]*#* Association was stronger for TET2: HR 2.5 [1.5–4.1]

The relevance of CHIP in HFpEF is further influenced by the comorbidity burden in this population. HFpEF is characterized by a high prevalence of comorbidities, which are associated with a pro-inflammatory state. T2D occurs in approximately 35% HFpEF patients and has been linked to an increased risk of developing CHIP. Individuals with both T2D and CHIP exhibit a greater burden of cardiovascular comorbidity than those with T2D alone [46]. Obesity has similarly been associated with a higher prevalence of CHIP, and clonal growth may be more pronounced in the presence of metabolic dysfunction and inflammation, although the evidence is limited [47–49]. Consistently, CHIP carriers with CRP levels higher than 2 mg/L demonstrated the highest risk of incident HFpEF, whereas CHIP in the absence of elevated CRP was not consistently associated with disease development [44]. These findings suggest that clonal hematopoiesis interacts with comorbidity-driven inflammation, amplifying systemic immune activation and thereby increasing susceptibility to HFpEF.

Importantly, CHIP has been correlated with structural and functional cardiac abnormalities. Individuals with CHIP mutations showed worse echocardiographic measures of diastolic function, including higher E/e’ and E/A ratios and shorter deceleration time, reflecting impaired ventricular relaxation and elevated filling pressures. Additionally, an increased left atrial volume index has been observed in HFpEF patients with CHIP, indicating more advanced structural remodeling compared with those without CHIP mutations [43].

Overall, current evidence indicates that CHIP mutations, particularly TET2-associated mutations, are enriched in HFpEF and linked to more advanced diastolic dysfunction and adverse clinical outcomes. These findings highlight clonal hematopoiesis as a relevant modifier of HFpEF susceptibility and progression, especially within inflammatory and metabolically driven phenotypes.

## Future Perspectives

### Chip as Biomarker in HFpEF

Genetic sequencing for detecting CHIP can be performed in any individual and has become increasingly affordable with advances in sequencing technologies such as whole genome sequencing. Incorporating CHIP as a biomarker in HFpEF aligns with the move toward precision medicine, with its main value lying not in diagnosis but in early detection, risk stratification, patient phenotyping, and guiding therapeutic decisions.

- Early detection of disease: CHIP is detectable before overt disease has developed, as it is associated with the incidence of heart failure and other diseases[7, 45]. In individuals with cardiovascular risk factors, CHIP may signal an increased risk of HFpEF development, which can be especially relevant in ageing populations and patients with the metabolic syndrome. CHIP might be used as a preclinical biomarker for early disease detection.
- Risk stratification: The few studies that investigated the influence of CHIP (mainly TET2) on the prognosis of patients with HFpEF showed a significant impact on heart failure hospitalization and mortality[43, 36]. Using CHIP as a biomarker could identify a high-risk HFpEF subgroup that might benefit from closer monitoring and intensified therapy.
- Patient phenotyping: HFpEF is a heterogeneous syndrome comprising multiple phenotypic subgroups. CHIP may help define a biologically distinct subtype characterized by inflammation, in which immune activation rather than purely hemodynamic factors drives disease progression. Previous phenotypic clustering efforts in HFpEF have identified three distinct phenoclusters with differing prognoses[50]. Incorporating CHIP into such analyses could group patients with higher cytokine levels and more pronounced endothelial dysfunction, thereby delineating a distinct molecular subgroup. The value of CHIP screening is likely the most valuable in older patients with a high inflammatory and/or metabolic burden. CHIP may increase inflammation in patients whose comorbidities, such as obesity or T2D, already drive an inflammatory state, further raising their risk of developing HFpEF. CHIP screening could therefore help identify these high-risk patients. In younger patients, the low prevalence of CHIP likely limits how efficient screening would be at a population level, even though the association with incident HFpEF appears to be driven by a distinct, DNMT3A-related mechanism. Similarly, in patients with prevalent cardiovascular disease, the main value of CHIP status may be more therapeutic than diagnostic.
- Therapeutic guidance: Since CHIP is related to inflammation, CHIP-positive patients may respond differently to specific anti-inflammatory therapies, but also to SGLT2-inhibitors. CHIP sequencing might therefore help to select patients for targeted therapies but can also enrich clinical trials. Additionally, there is potential for CHIP in monitoring disease progression and response to therapy by measuring changes in clone size and inflammatory markers.

Although the potential of CHIP as a biomarker in HFpEF is high, there are still logistical and scientific hurdles to overcome such as: (1) the infrastructure of CHIP sequencing, (2) to determine which variants matter most and which accompanied threshold for clone size is relevant, and the fact that (3) CHIP is not disease-specific (as it is also associated with other cardiovascular diseases such as atherosclerosis, stroke, dilated cardiomyopathy, HFrEF). While the number of studies remains still limited, the role of CHIP in the incidence of HFpEF is more pronounced compared to HFrEF [40]. However, causality still needs to be proven.

### CHIP as Treatment Target in HFpEF

The growing evidence implicating CH in the pathophysiology of HFpEF raises the question whether it may represent a modifiable therapeutic target. Given that CH contributes to myocardial remodeling through enhanced immune activation, including IL-1β, IL-6, TNF, NLRP3, or the JAK pathway, anti-inflammatory strategies could reduce this inflammatory burden and thereby modify cardiovascular outcomes. Support for this concept derives from a genetic sub-analysis of the CANTOS trial, in which the IL-1β neutralizing antibody canakinumab reduced the risk of major adverse cardiovascular events by 62% in patients with a history of myocardial infarction and elevated systemic inflammation carrying TET2 mutations compared with 7% in those without [37]. In addition, an IL6R coding variant associated with attenuated IL-6 signaling reduced the risk of incident cardiovascular events in individuals with CHIP, particularly non-DNMT3A mutations [51, 52]. Furthermore, colchicine, an inhibitor of IL1β/NLRP3-driven inflammation, attenuated the association between TET2 mutations and myocardial infarction, which was not observed for other genes [38]. Similarly, a study of the Low-Dose Colchicine 2 (LoDoCo2) trial in individuals with coronary artery disease demonstrated attenuated clonal growth after colchicine treatment in those with TET2 mutations [53]. These findings raise the possibility that colchicine could limit clone growth and thereby slow progression of heart failure in patients carrying TET2 mutations.

Beyond modulation of inflammatory signaling, epigenetic modifiers have been proposed to restore normal differentiation of mutant hematopoietic cells. CHIP-associated mutations frequently affect genes involved in DNA methylation and chromatin remodeling. Thus, pharmacological agents inhibiting aberrant methylation patterns or chromatic structures have been suggested as potential strategies to reduce clonal expansion [54]. However, they lack clone specificity and affect also healthy cells. Alternatively, vitamin C as a cofactor for TET enzyme activity has emerged as a potential epigenetic therapy for TET2-mutated CH. Although clinical data remain limited, vitamin C has shown feasibility in early CH and offers a relatively low-toxicity approach for targeting epigenetic dysregulation in TET2-mutants. Moreover, metabolic modulators may also be relevant as CH induces metabolic reprogramming, thereby upregulating mitochondrial oxidative phosphorylation pathways to maintain their competitive advantage. Indeed, clinical evidence showed that patients treated with an inhibitor of mitochondrial respiration, metformin, had approximately 50% lower prevalence of specifically the DNMT3A R882 mutations [55].

Directly eliminating or neutralizing mutant hematopoietic clones is another therapeutic option. Immune-based approaches aim to selectively target cells carrying CHIP mutations. Recurrent mutations can generate neoantigens that distinguish mutant blood cells from normal cells, thereby providing potential immunogenic targets. One approach involves chimeric antigen receptor T-cell therapy, in which a patient’s T-cells are modified to recognize and attack CH cells expressing specific antigens. Alternatively, bispecific T-cell engagers are proteins that can simultaneously bind to a T-cell and a mutant cell, leading to the destruction of CH cells. However, remaining CH specific surface antigens remains a major challenge as CH-driving mutations mainly affect intracellular regulators rather than surface proteins. In addition, genome editing with CRISPR-Cas 9 could represent another more invasive strategy to correct the mutations in CH cells.

With the decreasing costs of omics analyses (e.g., transcriptomics and proteomics), many biomarkers are identified associated with heart failure. However, the majority remains as a biomarker that could have diagnostic or prognostic value, with barely any markers that could function as a actionable therapeutic target. Currently, CH is most promising and investigated as a prognostic marker that could guide therapy. However, in addition to what is already discussed in the previous paragraph, CH can be a precursor to hematological malignancies for which chemotherapy is needed. Whether chemotherapy for the treatment of CH can prevent heart failure is currently being investigated in the COLLAR study (Chemotherapy to treat cardiac injury and lower cardiovascular risk). In contrast to many other biomarkers, CH holds the potential as a treatment target for which future clinical trials will be necessary. Designing cardiovascular outcome trials with CH will require an interdisciplinary effort, to integrate the complex CH biology into therapies [56].

## Conclusion

In conclusion, HFpEF is a complex and heterogeneous syndrome in which chronic low-grade inflammation plays a central role in disease initiation and progression. Emerging evidence identifies CHIP as a novel contributor to this inflammatory milieu, linking ageing, immune dysregulation, and cardiovascular disease. Both experimental and clinical studies suggest that CHIP, particularly *TET2* mutations, may causally contribute to myocardial remodeling, diastolic dysfunction, and adverse outcomes in HFpEF. Importantly, the impact of CHIP appears to be context-dependent, interacting with comorbidities and systemic inflammation to amplify disease risk. This supports the concept that CHIP is not merely a biomarker of ageing, but an active driver of pathophysiology in a subset of patients. Incorporating CHIP into clinical frameworks may improve risk stratification, enable more refined phenotyping, and guide therapeutic decision-making. At the same time, CHIP-associated inflammatory pathways represent promising targets for intervention, although disease-specific benefits remain to be established. Future studies should focus on validating these findings across diverse populations and clarifying mutation-specific effects. Larger prospective HFpEF studies should establish which driver genes and clone sizes carry clinical risk in this population. Interventional trials should also test whether the cardiovascular benefits seen with anti-inflammatory therapies in other CHIP-associated diseases also apply to HFpEF. In addition, further research should identify which patient populations are most likely to benefit from CHIP screening and anti-inflammatory therapies. Ultimately, integrating CHIP into HFpEF research may facilitate the transition toward more personalized and mechanism-based care.

## Key References

- Evans MA, Walsh K. The Immunological Consequences of Clonal Hematopoiesis in Heart Failure. Immunol Rev. 2026;337(1):e70094. doi:10.1111/imr.70094.
  - A detailed systematic review on the immunological consequences of CHIP in heart failure.
- Rhee JW, Bolton KL, Gupta D, Weeks LD, Bick AG, Tall AR et al. Clonal Hematopoiesis and Its Cardiovascular Implications: A Scientific Statement From the American Heart Association. Circulation. 2026;153(11):e940-e52. doi:10.1161/cir.0000000000001404.
  - Scientific consensus statement from the AHA on the current status of CHIP in cardiovascular diseases.
- Cochran JD, Yura Y, Thel MC, Doviak H, Polizio AH, Arai Y et al. Clonal Hematopoiesis in Clinical and Experimental Heart Failure With Preserved Ejection Fraction. Circulation. 2023;148(15):1165-78. doi:10.1161/circulationaha.123.064170.
  - Original human and murine study demonstrating a causal role for CHIP specifically in HFpEF.
- Schuermans A, Honigberg MC, Raffield LM, Yu B, Roberts MB, Kooperberg C et al. Clonal Hematopoiesis and Incident Heart Failure With Preserved Ejection Fraction. JAMA network open. 2024;7(1):e2353244. doi:10.1001/jamanetworkopen.2023.53244.
  - Cohort study demonstrating that CHIP, specifically TET2 mutations, are an independent risk factor for incident HFpEF.

## Data Availability

No datasets were generated or analysed during the current study.
